# Conversion of Unmodified Stem Cells to Pacemaker Cells by Overexpression of Key Developmental Genes

**DOI:** 10.3390/cells12101381

**Published:** 2023-05-13

**Authors:** Tahereh Karimi, Zhizhong Pan, Vladimir N. Potaman, Eckhard U. Alt

**Affiliations:** 1 Heart and Vascular Institute, Department of Medicine, Tulane University Health Science Center, 1430 Tulane Ave, New Orleans, LA 70112, USA; tara@cemvita.com; 2 Alliance of Cardiovascular Researchers, 2211 Augusta Dr #10, Houston, TX 77057, USA; 3 University of Texas MD Anderson Cancer Center, Houston, TX 77030, USA; 4 InGeneron Inc., 8205 El Rio Street, Houston, TX 77054, USA; 5 Sanford Health, University of South Dakota, Sioux Falls, SD 57104, USA; 6 Isar Klinikum Munich, Sonnenstr 24-26, 80331 Munich, Germany

**Keywords:** adipose-derived stem cells, transcription factors, ion channels, pacemaker cells

## Abstract

Arrhythmias of the heart are currently treated by implanting electronic pacemakers and defibrillators. Unmodified adipose tissue-derived stem cells (ASCs) have the potential to differentiate into all three germ layers but have not yet been tested for the generation of pacemaker and Purkinje cells. We investigated if—based on overexpression of dominant conduction cell-specific genes in ASCs—biological pacemaker cells could be induced. Here we show that by overexpression of certain genes that are active during the natural development of the conduction system, the differentiation of ASCs to pacemaker and Purkinje-like cells is feasible. Our study revealed that the most effective procedure consisted of short-term upregulation of gene combinations SHOX2-TBX5-HCN2, and to a lesser extent SHOX2-TBX3-HCN2. Single-gene expression protocols were ineffective. Future clinical implantation of such pacemaker and Purkinje cells, derived from unmodified ASCs of the same patient, could open up new horizons for the treatment of arrythmias.

## 1. Introduction

Cardiac rhythm problems (arrhythmias) manifest themselves in disturbed heartbeat and compromised blood circulation due to an abnormal function of pacemaker or Purkinje cells which secure the heart to contract normally [1]. Pacemaker dysfunction-related diseases include atrial and ventricular fibrillation, heart block, bradycardia-tachycardia syndrome, and sick sinus syndrome. The latter refers to arrhythmias in which the sinoatrial node (SAN) comprising pacemaker cells does not work properly. Coronary heart disease, myocardial infarction, cardiomyopathy, and genetic defects or infections, are causative, most often concomitant with aging [2,3]. Artificial pacemakers, medical implant devices which send electrical impulses delivered by electrodes to depolarize the heart muscle, are used to support an adequate heart rate [4,5,6]. The drawbacks of electronic pacemakers are general risks associated with the implant of a foreign body, the absence of hormonal response, possible interference from external magnetic fields, and limited battery life, etc. [5,6,7]. All of these prompted the search for potentially more effective biological alternatives. Biological pacemakers, generally intended as non-contractile myocardial cells to induce spontaneous activity in contractile cardiac tissue, could represent a potential tool to overcome the limitations of implantable electronic devices.

**Figure 1:**
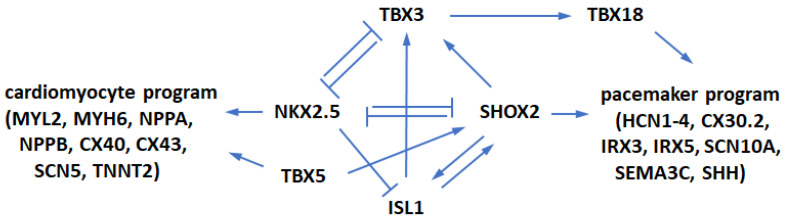
The regulatory network controlling SAN morphogenesis. A stimulatory-repressive regulatory network controls pacemaker cells and atrial working cardiomyocyte development.

To understand and create functional replacement pacemaker cells, it might be instructive to follow their origins and developmental pathways in the heart. During embryonic development, the population of early cardiac progenitor cells is formed which expresses core cardiac transcription factors (TFs) including GATA4, HAND2, ISL1, MEF2C, NKX2.5, and TBX5 [8]. During embryonic development, cells split their developmental pathway and specialize into contractile cardiomyocytes and non-contractile electrical cells, namely, pacemaker and Purkinje cells. Atrial and ventricular cardiomyocytes continue expressing TF NKX2.5 and subsequently express contractile factors MYL2, MYH6/α-MHC, and MYH7/β-MHC among others [9]. On the other hand, in pacemaker cells, TF SHOX2 inhibits NKX2.5 expression [10] and activates pacemaker genes including TF TBX3 and channel proteins HCN1-4. This is the point in heart development where the pathway to contractile heart muscle cardiomyocytes and to non-contractile Purkinje and pacemaker cells splits. Figure 1 schematically shows the relationships between key relevant genes. The T-box TF TBX3 suppresses contractile myocardial differentiation in the developing SAN and allows these cells to acquire the non-contractile electrical phenotype. Importantly, other T-box TFs are also involved in the pacemaker cell development, although their precise roles have not been fully understood [10,11,12]. For example, the level of TBX5 expression positively controls the levels of TFs SHOX2 and TBX3 in the developing heart [13], which is important for the initiation of SAN formation. TBX5 is necessary beyond initiation stages because its expression continues in TBX5^+^/SHOX2^+^/NKX2.5^−^ SAN cells [14,15]. TF TBX18 is also important in SAN development by controlling the formation of the SAN head from mesenchymal precursors, on which TBX3 subsequently imposes the pacemaker gene program [16]. At later stages, TBX18 is expressed in the SAN head but not in the SAN tail [14]. Thus, several TFs (SHOX2, TBX3, TBX5, and TBX18) and channel proteins HCN1-4 provide critical contributions to the formation of SAN in vivo.

Similar to reprogramming cells of other types, different options in replenishing lost pacemaker cells using multipotent cells or cells of lineages developmentally close to pacemakers have been studied. Gene expression in situ by direct gene transfer into the heart or in vitro by treating cells with lineage-specific factors in culture for subsequent transplantation into the heart may enforce a developmental program driving the generation of pacemaker cells from other cell types.

Several channel protein genes (HCN1, HCN2, HCN4, SKM1, KCNN4) and genes of transcription factors (TBX3, TBX18) that are active during the genesis of the pacemaker system have been used by researchers in numerous efforts to develop an optimal pacemaker generation protocol [17,18,19,20,21,22,23,24,25,26,27,28,29,30,31,32,33,34,35,36,37,38,39,40,41,42,43,44]. The pacemaker system represents a complex structure whose function is regulated by many factors and multiple genes. It is tempting to search for a single factor that might be dominant enough to define the crucial parameters of the entire system. Yet, so far, stem or other cell programming efforts with single factors have not yet achieved full physiological functionality or morphological properties of natural cardiac pacemaker cells. It should be noted that in a similar case, transdifferentiation of cells committed to other lineages into cardiomyocytes was uneasy and required a combination of lineage-specifying factors [45,46,47]. Therefore, it might have been unreasonable to expect highly efficient pacemaker derivation by expression of single genes. Several studies showed that combinations of programming factors (HCN2 + SKM1, TBX3 + HCN2, TBX18 + ISL1) may result in better conversion efficiency and/or the baseline and maximal beating rates closer to the optimal biological pacemaker range than provided by the same separate genes [20,38,39,41].

Adult adipose tissue-derived stem cells (ASCs), a subtype of the recently defined vascular-associated pluripotent stem cells (vaPS cells [48,49]), are highly promising candidates for pacemaker reprogramming. They possess the important potential for differentiation into all three germ layers. One of the well-known MSC sources is bone marrow (BM). Several studies have used BM-MSCs for conversion into pacemaker-like cells [17,19,30,32,36]. However, multipotent MSCs only comprise a minor fraction of BM cells, whereas the bulk of BM are hematopoietic progenitors, wrongly and incorrectly often considered as true stem cells. BM-MSCs typically need to be expanded in culture to obtain adequate numbers before pacemaker conversion. As such, they are known to rather quickly lose their telomere ends when passaged in vitro [50]. This likely results in their inferior regenerative properties compared with ASCs [51]. Several studies with ASCs on their cardiac differentiation path described pacemaker derivation induced by transcription factor or channel protein genes [27,28,31,33,39,42].

A head-to-head comparison showed an earlier onset of spontaneous contractions and higher beating regularity, synchrony, and rate in transgene-stimulated ASCs than in BM-MSCs [33]. MSCs are abundant in adipose tissue, which yields significantly more MSCs per unit volume than BM [48,49,52,53]. Autologous stem cells may be easily obtained and amplified, have no immunogenic potential, and can be differentiated into several cell types, such as adipocytes, osteoblasts, hepatocytes, and neurons [31,48,49,54]. From a practical standpoint, for regenerative medicine in humans, the preferred source of adult multipotent stem cells are autologous cells, such as adipose-derived regenerative cells (ADRCs) [49], also named stromal vascular fraction (SVF), from the same patient who is in need of a cure for arrythmia. Transcriptome analysis of MSCs at a single-cell level indicated that upon passaging “adherent culture supports development of fibroblast-like phenotype” [55]. Thus, using unmodified (minimally manipulated) ASCs may be advantageous for retaining their differentiation potential.

Here we tested combinations of transcription factors SHOX2, TBX3, TBX5, and TBX18, and channel protein HCN2 for conversion of ASCs toward pacemaker cells. To single out the effects of specific factors on the initial stages of this process, gene expression was induced for a relatively short time of three days. Inducible expression of lentivirally delivered genes in ASCs was followed by cell incubation during which changes in cellular morphology, pacemaker gene expression, and development of funny currents served to monitor reprogramming.

## 2. Materials and Methods

### 2.1. Adipose-Derived Stem Cells

Human unmodified fresh ADRCs were isolated at InGeneron (Houston, TX, USA) as described previously [54]. Adipose tissue was obtained via lipoaspiration from 30–40-year-old subjects undergoing elective lipoplasty with informed consent according to the IntegReview IRB approved protocol #200601001 (IntegReview IRB, Austin, TX, USA). After addition of the Matrase Reagent (a GMP-certified, proprietary enzyme blend of collagenase and neutral protease), 25 mL lipoaspirate was processed in the InGeneron Transpose RT centrifuge-based processing unit. The processed lipoaspirate solution was filtered through a 200-μm filter, washed with saline, and the cells were separated from the rest of the tissue by centrifugation at 600 g for 5 min at room temperature. Then, the cell fraction containing concentrated ADRCs (ca. 2 mL) was extracted through a swabable luer vial adapter at the bottom of the wash tube, and the remaining substances (fat, cellular debris, and liquid) were discarded. The wash-centrifugation step was repeated, and the final cell suspension was collected into 3 mL saline. For expansion, cells were pelleted and finally suspended in growth media containing α-MEM (Corning, Glendale, AZ, USA), 20% FBS (Atlanta Biologicals, Flowery Branch, GA, USA), 2 mM glutamine, and 100 units/mL penicillin with 100 μg/mL streptomycin (Corning). Adherent cells (ASCs) were grown in culture flasks at 37 °C in a humidified atmosphere containing 5% CO_2_, followed by daily washes to remove red blood cells and non-attached cells. For passaging, at ca. 80% confluence, cells were detached using 0.25% trypsin solution and seeded at the density of 3000 cells/cm^2^ in fresh cell culture plates. Cells of passages 3–5 were used for subsequent pacemaker conversion.

### 2.2. Lentivector Preparation

The parent plasmid for transcription factor gene cloning was a bicistronic doxycycline-inducible gene expression lentivector [56]. The gene-coding sequences of TFs SHOX2, TBX3, TBX5, TBX18 and the hyperpolarization-activated cyclic nucleotide-gated channel protein HCN2 were separately cloned into the same parent vector downstream of the seven tetracycline-responsive elements (binding its stable analog, doxycycline, Dox) and the minimal CMV promoter. All pDox-transgene plasmids were validated by sequencing. These plasmids, the plasmid for reverse Tet transactivator (rtTA2) and packaging plasmids psPAX2 (Addgene plasmid #12260) and pMD2.G (Addgene plasmid #12259) were propagated in Stbl3 E. coli cells (Invitrogen, Carlsbad, CA, USA), extracted with the Qiagen Maxiprep kit, and quantified with the NanoDrop spectrophotometer.

### 2.3. Lentiviral Production

Production of lentiviruses for reprogramming transgenes was carried out in HEK-293T cells. Briefly, 10 µg of transfer plasmid (pDox-transgene or rtTA2), and 5 µg each of psPAX2 and pMD2.G were transfected into confluent HEK-293T cells on a 100-mm plate using FugeneHD (Promega, Madison, WI, USA). Viral supernatant was collected after 48 and 72 h, filtered through a 0.45-μm filter, and concentrated using the Lenti-X Concentrator solution (Clontech, Mountain View, CA, USA) according to the manufacturer’s guidelines. The resulting supernatant was removed, and the remaining virus-containing pellets were suspended in phosphate-buffered saline (PBS). The virus was stored in single-use aliquots at −80 °C.

### 2.4. Cell Culture

For pacemaker conversion, ASCs were cultured in α-MEM (Invitrogen) supplemented with 5% (*v*/*v*) horse serum, 0.1 mM non-essential amino acids (Gibco, Waltham, MA, USA), 2 mM L-glutamine, and 100 U/mL streptomycin/penicillin [57]. The cell media was changed every other day. To differentiate ASCs into cardiac pacemaker cells, 80% confluent ASCs were infected with different combinations of the viral particles containing SHOX2, TBX3, TBX5, TBX18, and HCN2 vectors according to the predefined experimental groups (Table 1). Cultures were treated daily with 400 ng/mL Dox for 3 days, then sustained for 2 weeks in α-MEM with above-mentioned supplements. Daily microscopic observation of cells was performed using a Nikon Ti-E inverted microscope equipped with a DS-Fi1 5 M color camera to study the morphological changes of cells after the transfection. RNA samples were collected from different experimental groups on day 14 after the initiation of Dox induction.

### 2.5. Immunohistochemistry (IHC)

IHC staining for the major marker proteins of cardiac pacemaker cell lineages including CX30.2 and HCN4 were performed 2 weeks after the initiation of doxycycline induction. SHOX2-TBX5-HCN2 treated ASCs were washed with PBS, permeabilized with 0.1% Triton-X and reacted with HCN4 or CX30.2 primary antibodies (Abcam, Cambridge, MA, USA) and Alexa Fluor 594 or Alexa Fluor 647 secondary antibodies (Abcam). Nuclei were counterstained with 4′,6-diamino-2-phenylindol (DAPI).

### 2.6. Cell Sorting by Size

After two weeks in culture, converted cells were washed twice with PBS, detached from the plates with 0.25% trypsin, neutralized with FBS and pelleted in a centrifuge. Cells were then reconstituted to 10^6^ cells/mL in α-MEM and sorted by size in a FACSAria II flow cytometer using a forward scatter detection.

### 2.7. qRT-PCR

To evaluate the effects of various combinations of cardiac pacemaker-inducing factors on ASC differentiation toward cardiac conduction cell lineage, mRNA expression levels of specific marker genes in both cell lineages were analyzed by qPCR. The genes of interest included late-stage markers of cardiac pacemaker cells HCN3, HCN4, KCNN4, and CX30.2. Total RNA was isolated using the Qiagen RNeasy Kit and then reverse-transcribed into cDNA using SuperScript III reverse transcriptase (Invitrogen). Quantitative PCR was performed with the ABI Prism 7000 System Detection Sequence and software (Applied Biosystems, Waltham, MA, USA) using SYBR Green (Applied Biosystems) for detection.

### 2.8. Patch Clamp Electrophysiology

The electrophysiological properties of genetically programmed pacemaker cells were measured via single-cell patch clamping and funny channel density two weeks after the initiation of transfection. Whole cell voltage-clamp experiments were carried out using the standard patch-clamp method [58]. Thin-walled borosilicate glass (1.5-mm, No. 7052, Garner Glass, Claremont, CA, USA) was used for pulling recording electrodes, using a Flaming/Brown microelectrode puller (P-97, Sutter Instruments, Novato, CA, USA), which were heat polished before use. The pipettes filled with internal solution had a tip resistance of 2–5 MΩ. Recordings were performed with an Axoclamp 2B patch-clamp amplifier (Axon Instruments, Foster City, CA, USA). The data filtered at 2 kHz were processed with Clampex 8 software (Axon Instruments). For current-clamp recordings, the intercellular solution contained 10 mM NaCl, 130 mM potassium aspartate, 0.04 mM CaCl_2_, 3 mM Mg-ATP, and 10 mM HEPES. The pH was adjusted to 7.2 with KOH. The extracellular bath solution contained 140 mM NaCl, 5.4 mM KCl, 1.8 mM CaCl_2_, 1 mM MgCl_2_, 10 mM glucose, and 5 mM HEPES at pH 7.4.

## 3. Results

### 3.1. Single Factors TBX3 and TBX5 and Their Combinations with SHOX2 and HCN2 Quickly Induce ASC Differentiation

**Figure 2:**
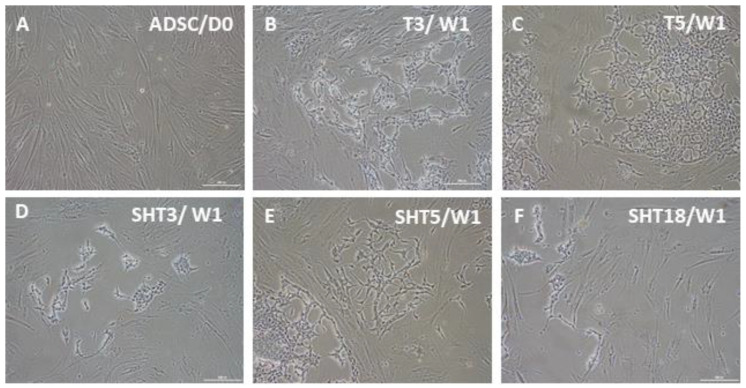
Bright-field microscopy of ASCs one week after doxycycline-induced three-day expression of pacemaker-specific genes. (**A**) Control, untransduced ASCs; (**B**) TBX3 (T3) only; (**C**) TBX5 (T5) only; (**D**) SHOX2-HCN2-TBX3 (SHT3); (**E**) SHOX2-HCN2-TBX5 (SHT5); (**F**) SHOX2-HCN2-TBX18 (SHT18). The appearance of ASCs after expression of SHOX2, HCN2, and TBX18 was very similar to that of untreated cells. Scale bar, 200 μm.

As a step toward the development of pacemaker cells from autologous adult stem cells and subsequent patient treatment, we used human ASCs which were transduced with lentiviruses for inducible expression of SHOX2 and several T-box transcription factors as well as the channel protein HCN2. Beginning from week two, after three-day doxycycline-induced gene expression, cells from different groups started to change their fibroblast-like morphology. The extents of such changes depended on specific factors and their combinations. Little change in cell morphology from the original ASCs (Figure 2A) was observed after expression of the single genes SHOX2, HCN2, and TBX18 as well as gene combinations SHOX2-HCN2 and SHOX2-HCN2-TBX18 (not shown). At the same time, expression of single factors TBX3 and TBX5 (Figure 2B,C), and SHOX2-HCN2 combinations with T-box factors (Figure 2D–F) induced noticeable but variable changes in cell appearance. For example, Figure 2E shows that cells, in which the gene combination SHOX2-HCN2-TBX5 was expressed, started forming clusters containing network-forming spindle-shape cell types. Expression of other T-box factors resulted in smaller changes: a less developed network structure formed in the cells that expressed TBX3 and SHOX2-HCN2-TBX3, whereas an even lesser change was observed in the SHOX2-HCN2-TBX18 cell group.

### 3.2. SHOX2-HCN2-TBX5 Is the Most Efficient Combination

**Figure 3:**
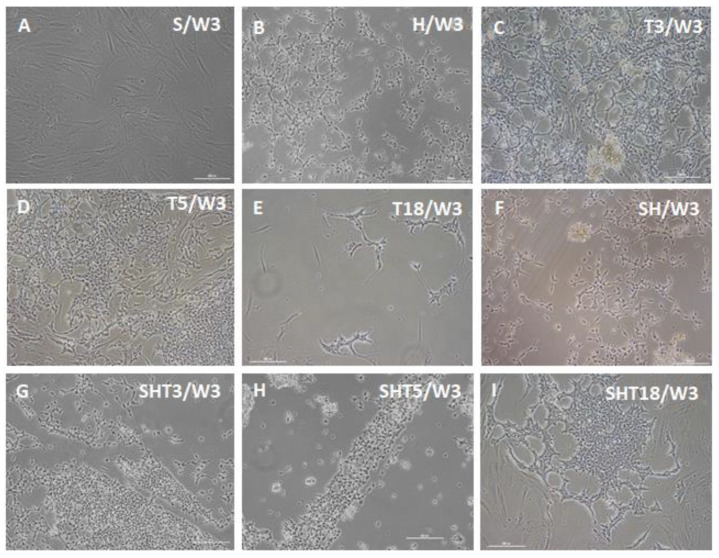
Bright-field microscopy of ASCs three weeks after doxycycline-induced three-day expression of pacemaker-specific genes. (**A**) SHOX2 (S) only; (**B**) HCN2 (H) only; (**C**) TBX3 (T3) only; (**D**) TBX5 (T5) only; (**E**) TBX18 (T18) only; (**F**) SHOX2-HCN2 (SH); (**G**) SHOX2-TBX3-HCN2 (SHT2); (**H**) SHOX2-TBX5-HCN2 (SHT5); (**I**) SHOX2-TBX18-HCN2 (SHT18). Scale bar, 200 μm.

During the following differentiation period, cell morphology changes started to develop in other gene expression groups. Figure 3 shows that similar cell morphologies may be induced by different factor sets, although within different timeframes. This is consistent with the results of previous studies, indicating that expression of various genes promoted conversion of several types of starting cells toward possibly similar pacemaker-like cells [18,19,20,21,22,23,24,25,26,27,28,29,30,31,32,33,34,35,36,37,38,39,40,41,42,43,44]. Observation of unmodified ASC cultures at one week (Figure 2), two weeks (not shown), and three weeks (Figure 3) indicates a more robust change in cell morphology for SHOX2-TBX5-HCN2 combination followed by the SHOX2-TBX3-HCN2 mix. Combinations including TBX18 (SHOX2-TBX18-HCN2) and lacking T-box factors (SHOX2-HCN2), as well as single factors TBX3 and TBX5, could also drive the morphology changes of ASCs towards pacemaker-like cells, albeit on a longer time scale.

### 3.3. SHOX2-HCN2-TBX5 Converted Cells Express Pacemaker Factors CX30.2 and HCN4

**Figure 4:**
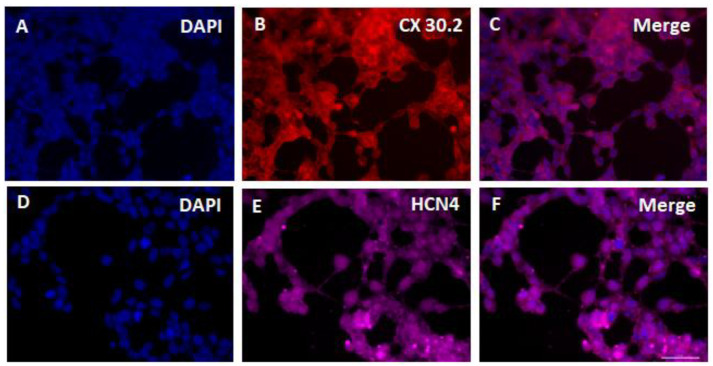
Immunohistochemistry of ASCs two weeks after doxycycline-induced three-day expression of SHOX2-TBX5-HCN2 genes using antibodies to major marker gene products of cardiac pacemaker cell lineages CX30.3 and HCN4. DAPI, 4′,6-diamino-2-phenylindole. Scale bar, 50 μm.

The IHC analysis shows that SHOX2-HCN2-TBX5 converted cells express pacemaker-specific markers CX30.2 and HCN4 after three weeks in culture (Figure 4). It should be noted that the induced three-day gene overexpression driving ASC conversion was only temporary, and normally the overexpressed proteins vanish in one or two days after the inducer is withdrawn. Thus, the IHC images demonstrate the sustained endogenous expression of CX30.2 and HCN4 without the supporting influence of reprogramming gene expression.

### 3.4. Two Types of Converted Cells Have Pacemaker-Like Characteristics

**Figure 5:**
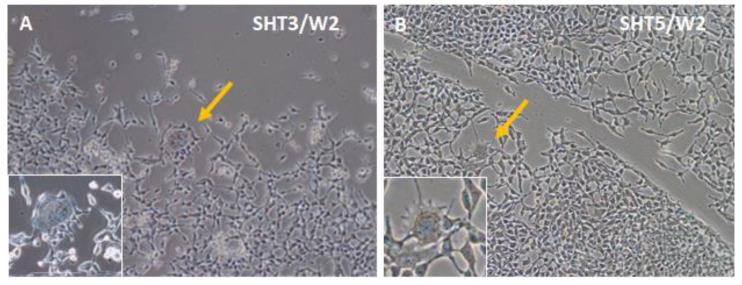
Observation of two different cell types in ASCs two weeks after (**A**) SHOX2-TBX3-HCN2 (SHT3), or (**B**) SHOX2-TBX5-HCN2 (SHT5) three-day expression. While the bulk of cells on plates had spindle shapes, the spider-shape cells are shown with yellow arrows and in magnified insets.

Figure 5 shows that during the differentiation period, numerous spindle-shaped cells with a relatively thicker (ca. 20 μm) central part and long projections (the majority of cells in Figure 5A,B) dominated the SHOX2-TBX3-HCN2 and SHOX2-TBX5-HCN2 cultures. They were similar to one type of isolated sinoatrial node cells [22,59]. Among spindle-shape cells, rare, spider-shape ones of more than 50-μm sizes (Figure 5A,B, indicated by arrows and shown in insets) were also detected. Their morphologies were reminiscent of another subtype of cells isolated from the sinoatrial node described earlier [22,60].

**Figure 6:**
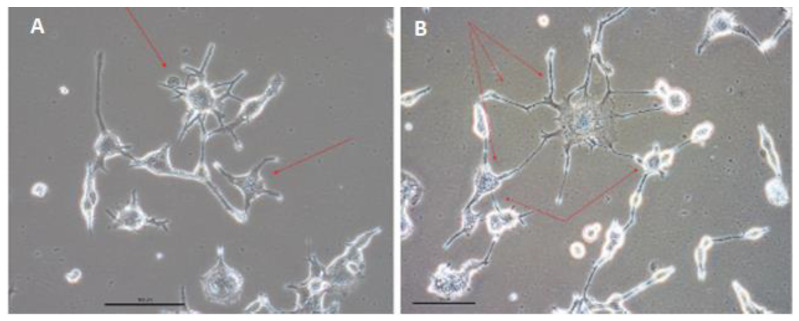
The spider-shape cells (indicated with red arrows) start to form networks with each other and with spindle-shape cells within three weeks after SHOX2-TBX5-HCN2 three-day expression (**A**,**B**). Different parts of the observation field are shown. Scale bar, 50 μm.

Subsequently, both populations of spindle- and spider-shape cells appeared to form interconnected networks with each other (Figure 6). In addition, small, spiked cells started to form highly aligned growth patterns: for example, a groove formation in Figure 3G, or a belt formation in Figure 3I.

**Figure 7:**
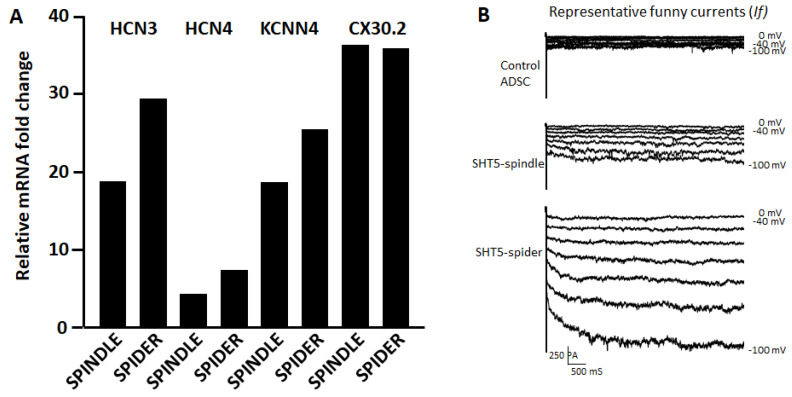
Analysis of spindle- and spider-shaped cells. (**A**) The expression levels of several pacemaker marker genes were quantified by qRT-PCR after size-sorting of SHOX2-HCN2-TBX5 converted cells. (**B**) Representative funny currents (*If*) recorded from untreated ASCs (control) cells, from small size fraction (spindle cell size ca. 20µm) and large size fraction (spider cell size > 50 µm) of SHOX2-TBX5-HCN2 transfected ASCs. The *If* currents were recorded with voltage steps from −100 mV to −40 mV for 500 mS with 10 mV increment from a holding potential of −40 mV.

The sizes of the two converted cell types were different enough to be separated by flow cytometry using forward scatter detection. The large cells comprised approximately 3% of the total populations. Analysis of relative mRNA expression (Figure 7A) indicates that both cell types express non-contractile cell-specific ion channels HCN3, HCN4, KCNN4, and CX30.2. Patch clamp recordings of currents in isolated single cells showed distinctly different levels of electrophysiological activity in smaller and in larger cells (Figure 7B). The funny currents from spider-shaped cells shown in Figure 7B are very similar to funny currents of cardiac pacemaker cells presented in the literature. See, e.g., Figure 4 in Ref. [22] and Figure 4 in Ref. [58].

## 4. Discussion

### 4.1. Unmodified ADRCs Containing Multipotent ASCs May Be Used as Starting Material for Forward Programming of Pacemaker-Like Cells

Forward programming of pacemaker-like cells for therapeutic purposes needs a reliable and easily accessible source of cells amenable to conversion. Adipose tissue is readily available from patients requiring possible treatment and provides an abundant supply of MSCs [31,54]. This makes it a preferred source of multipotent stem cells over bone marrow. Considering other types of stem cells, embryonic stem cells (ESCs) have limited use because of ethical issues [61], and ESCs and induced pluripotent stem cells (iPSCs), which might also be used for pacemaker derivation, have certain concerns regarding malignancy when used for therapeutic applications [62,63]. Combinations of two expressed factors have been recognized as inducing larger and faster cell changes towards the desired lineage compared with the single-factor effects [20,38,39,41].

We made a step forward in developing the pacemaker conversion strategy by using unmodified ASCs as the starting material and tested several combinations of pacemaker programming factors. Note that some trends observed in programming these cells were different from those reported for cardiac-like starting cells, because of the differences in the initial transcription profiles.

Unmodified ASCs have a typical fibroblast-like morphology, although sometimes cells reminiscent of the spindle-shaped ones were observed. During induced conversion, most cells adopt the spindle shape. HCN2 expression was able to induce the ASC conversion in culture, similar to previously tested HCN2 expression in MSCs following transplantation to the sites of SAN damage [17,20] or in cultured cardiac-like cells [24,25,33,37]. SHOX2 alone was not efficient in converting ASCs into differentiating cells [23,26]. One possible reason is that it normally inhibits NKX2.5 levels in cells that reached or passed the developmental stage of cardiac progenitors [10]. Inducing SHOX2 expression alone in ASCs may not involve NKX2.5 upregulation, therefore the role of SHOX2 has no significance. TBX18 was also not efficient in ASC conversion, contrary to previous reports where cells expressed TBX18 during their differentiation [27,30,32,34,35,36,39,44]. Its expression did not work alone, and its contribution as the part of SHOX2-TBX18-HCN2 set was relatively small (compare with SHOX2-HCN2 at three weeks). Apparently, for ASC conversion, it needs support from other genes.

T-box factors TBX3 and especially TBX5 provided pronounced enhancement of such conversion. Whereas TBX3 was used in several studies for differentiated and partially differentiated cells [18,22,40,41], here we show that it can be used for programming stem-like cells. TBX5 use in combination with other factors was also described for cardiac progenitors [42], but here we successfully used it for unmodified ASC cells. It should be noted that we observed some similar trends in developing properties of pacemaker-like cells to those reported by Raghunathan et al. [42]. However, ASC conversion lagged that of cardiac progenitors by approximately one week. Previously, TBX5 was part of the recipes used for cell transdifferentiation into cardiomyocytes [45,46,47] and it likely stimulated NKX2.5 expression. In the case of ASCs, TBX5 expression potentially contributes to their cardiac differentiation making them somewhat closer to NKX2.5-expressing cardiac and cardiac-like cells used by other researchers [21,24,25,29,35,37,38,41,42,43,44]. Furthermore, TBX5, which positively regulates the levels of SHOX2 and TBX3 in the developing heart, may induce the expression of these pacemaker lineage genes during ASC conversion. The SHOX2-TBX5-HCN2 set was the most efficient, but TBX5 itself was to some extent also capable of cell conversion. It was potentially because TBX5 stimulates SHOX2 and then TBX3 (Figure 1) which are needed for pacemaker conversion. It is not clear how strongly the TBX5-upregulated SHOX2 opposes NKX2.5 upregulation also brought up by TBX5. However, it seems that the summary effect of TBX5 on expression of discussed genes is beneficial for pacemaker cell development from ASCs.

### 4.2. Cell Identities

The tested combinations of factors, SHOX2, with either TBX3 or TBX5 and HCN2, induced ASC conversion, which resulted in mostly smaller spindle-type cells, with the large spider-shape cells as a minor fraction. In both cell types, ion channel properties, such as expression of cardiac channel genes and in the larger cells of funny currents, were detected. The two cell types that we observed have been previously described [22,59,60,64,65]. They had overlapping gene expression profiles and electrophysiological properties. It may not yet be completely conclusive to classify them, although in our work the spindle-shape cells formed aligned structures (grooves and belts), which likely represent immature Purkinje fibers. Furthermore, the spindle-shape cells were less electrophysiologically active than the larger spider-shape ones that express the funny ion current, consistent with the properties of pacemaker cells. Note that their size and shape (50-μm diameter, plus their radiating protrusions) are not present in any other somatic cell type in the body [65].

### 4.3. ASCs as the Cells of Choice for Pacemaker Programming

Adipose tissue is readily available from most patients without possible discomfort associated with tapping bone marrow sources or using allogenic cells—obviating possible rejection issues—which shows their advantage over embryonic stem cells. We have been long involved in translational studies of adipose-derived cells in regenerative cell therapy [48,49]. Freshly isolated, uncultured, autologous adipose-derived regenerative cells (ADRCs), also often named stromal vascular fraction (SVF), have emerged as a promising tool for tissue regeneration [49,54,66,67]. Human adipose tissue obtained during liposuction contains mature adipocytes and easily extractable ADRCs or SVF. The latter are a rich source of ASCs capable of adipogenic, osteogenic, hepatogenic, and neurogenic differentiation, that is, capable of differentiating into cells of all three germ layers [54]. Here we showed their ability to convert into pacemaker and Purkinje-like cells. One may envisage the potential application of such pacemaker cells, not only for patients with known familial history of cardiac arrhythmias but in general for patients in need for an initial pacemaker implantation or a replacement of an implanted pacemaker at the end of its battery life. If the implantation of ASC-derived Purkinje cells proves to be beneficial for treatment of re-entry tachyarrhythmias caused by slow conduction in ischemic and/or fibrotic myocardium, it should be evaluated by further studies. If such a novel approach is clinically successful, it may potentially change the practice of medicine by shifting from the implantation of pacemaker and defibrillator devices to a “natural healing” using the body’s own regenerative power.

### 4.4. Choice of Gene Delivery Vehicle

One approach to the pacemaker gene therapy is forward programming of ASCs to a pacemaker lineage using the patient’s own autologous cells in an essentially ex vivo procedure followed by subsequent transplantation of modified cells. For this, the lentiviral delivery of programming genes is easily achieved, perhaps in combination with a selection step (either drug selection or FACS sorting). Such lentiviral cell treatment may be accomplished with constitutively expressed dominant pacemaker genes or after transient gene expression as conducted in our study. Injection of pacemaker cells into SAN would restore the heart rhythm by cells that would not further divide thus preventing the expansion of cells with integrated gene sequences at the fragile chromosome sites. As an alternative, mRNA-based reprogramming may be used [68]. A recent mRNA vaccination campaign against COVID-19 demonstrated that such an approach is feasible. The treatment of ASCs may be performed by repeated addition of reprogramming factor-encoding linear mRNA for several days, which would be similar to treatment with induced gene expression from lentivirus. Otherwise, the self-replicating [69] and circular RNA [70] may be used allowing a reduction in treatment frequency or limiting it to one-time RNA addition to cell culture.

## Figures and Tables

**Table 1:** List of experimental groups by controlled expression of single, double, and triple combinations of pacemaker inducing factors in ASCs.

Experimental Group Name	Description of Treatments
SHOX2	ASCs transduced with SHOX2
TBX3	ASCs transduced with TBX3
TBX5	ASCs transduced with TBX5
TBX18	ASCs transduced with TBX18
HCN2	ASCs transduced with HCN2
SH	ASCs transduced with double combination of SHOX2 and HCN2
SHT3	ASCs transduced with triple combination of SHOX2, TBX3 and HCN2
SHT5	ASCs transduced with triple combination of SHOX2, TBX5 and HCN2
SHT18	ASCs transduced with triple combination of SHOX2, TBX18 and HCN2

## Data Availability

The data presented in this study are available upon request from the corresponding author.

## Glossary

A cellular preparation that results from culturing fresh, unmodified Adipose-Derived Regenerative Cells (ADRCs) is called adipose-derived stem cells (ASCs).
